# A multi-center, open-label trial to compare the efficacy and pharmacokinetics of Artemether-Lumefantrine in children with severe acute malnutrition versus children without severe acute malnutrition: study protocol for the MAL-NUT study

**DOI:** 10.1186/s12879-015-0963-3

**Published:** 2015-06-12

**Authors:** Lise Denoeud-Ndam, Alassane Dicko, Elisabeth Baudin, Ousmane Guindo, Francesco Grandesso, Issaka Sagara, Estrella Lasry, Pedro Pablo Palma, Angeles M. Lima Parra, Kasia Stepniewska, Abdoulaye A. Djimde, Karen I. Barnes, Ogobara K. Doumbo, Jean-François Etard

**Affiliations:** Epicentre, Paris, France; Malaria Research and Training Center, Faculte de Médecine, Pharmacie et d’Odonto-stomatologie, Université des Sciences Techniques et Technologies de Bamako, Bamako, Mali; Medecins sans Frontieres, Paris, France; Medecins sans Frontieres, Barcelona, Spain; Worldwide Antimalarial Resistance Network (WWARN), Oxford, UK; Centre for Tropical Medicine, Nuffield Department of Clinical Medicine, University of Oxford, Oxford, UK; Division of Clinical Pharmacology, University of Cape Town, Cape Town, South Africa; TransVIHMI UMI 233, Institut de recherche pour le développement (IRD) – Inserm U 1175 – Montpellier 1 University, Montpellier, France

**Keywords:** Malaria, Severe acute malnutrition, Artemether-lumefantrine fixed combination, Pharmacokinetics, Efficacy, Niger, Mali

## Abstract

**Background:**

Malnutrition and malaria frequently coexist in sub-Saharan African countries. Studies on efficacy of antimalarial treatments usually follow the WHO standardized protocol in which severely malnourished children are systematically excluded.

Few studies have assessed the efficacy of chloroquine, sulfadoxine-pyrimethamine and quinine in severe acute malnourished children. Overall, efficacy of these treatments appeared to be reduced, attributed to lower immunity and for some antimalarials altered pharmacokinetic profiles and lower drug concentrations. However, similar research on the efficacy and pharmacokinetic profiles of artemisinin-combination therapies (ACTs) and especially artemether-lumefantrine in malnourished children is currently lacking.

The main objective of this study is to assess whether artemether-lumefantrine is less efficacious in children suffering from severe acute malnutrition (SAM) compared to non-SAM children, and if so, to what extent this can be attributed to a sub-optimal pharmacokinetic profile.

**Methods/design:**

In two sites, Ouelessebougou, Mali and Maradi, Niger, children with uncomplicated microscopically-confirmed *P. falciparum* malaria aged between 6 and 59 months will be enrolled. Two non-SAM children will be enrolled after the enrolment of each SAM case. Children with severe manifestations of malaria or complications of acute malnutrition needing intensive treatment will be excluded.

Treatment intakes will be supervised and children will be followed-up for 42 days, according to WHO guidance for surveillance of antimalarial drug efficacy. Polymerase Chain Reaction genotyping will be used to distinguish recrudescence from re-infection. SAM children will also benefit from the national nutritional rehabilitation program.

Outcomes will be compared between the SAM and non-SAM populations. The primary outcome will be adequate clinical and parasitological response at day 28 after PCR correction, estimated by Kaplan-Meier analysis. To assess the pharmacokinetic profile of lumefantrine, a sparse sampling approach will be used with randomized allocation of sampling times (5 per child). A total of 180 SAM children and 360 non-SAM children will be recruited during the 2013 and 2014 malaria seasons.

**Discussion:**

This study will provide important information that is currently lacking on the effect of SAM on therapeutic efficacy and pharmacokinetic profile of artemether-lumefantrine. If it shows lower therapeutic efficacy and decreased lumefantrine concentrations, it would inform dose optimization studies in SAM children.

**Trial registration:**

ClinicalTrials.gov: NCT01958905

## Background

Malnutrition and *Plasmodium falciparum* malaria are two major public health problems in sub-Saharan Africa. In Sahel countries, both diseases have a marked seasonality, with concomitant peaks in the second half of the year. The rainy season, roughly from July to October, corresponds with the high transmission season of malaria, and to the hunger gap period. In these countries, children under 5 years of age are the highest-risk population for both diseases, and it has been estimated that more than 50 % of child deaths were attributable to malnutrition potentiating effects on infections [1, 2].

Child malnutrition is a diverse spectrum: stunting i.e. chronic malnutrition reflected by a low height-for-age, and wasting i.e. acute malnutrition reflected by a low weight-for-height. The latter allows detecting acute or sub-acute episodes, and is a good predictive marker of short-term mortality. It is an indication to refer children into nutritional rehabilitation programs [3], together with the measurement of mid-upper arm circumference (MUAC). Anthropometric standards have been updated by the WHO in 2006 [4]. Moderate acute malnutrition (MAM) is defined by a weight-for-height z-score between -3 and -2, or MUAC between 115 and 125 mm, whereas severe acute malnutrition (SAM) is defined by z-score < -3or MUAC <115 mm.

Child malnutrition is associated with a higher risk of infections and infectious episodes contribute to deteriorate the nutritional status [5], resulting in a vicious circle malnutrition-infection-malnutrition. In the specific context of malaria infections, the interactions with malnutrition are complex. The question of the impact of child malnutrition on malaria susceptibility is still debated, with conflicting results in the literature: few studies have found an increased risk of malaria and higher parasitemia in children with stunting, linked to decreased specific antimalarial immunity [6, 7]. Other studies have shown a higher risk of malaria-related severe anemia in wasted children [8, 9] and stunted children [10]. Overall, children with low weight/age are at higher risk to develop malaria in general, and severe episodes in particular [9, 11], with lower parasitemia than in non-malnourished children [12]. It is also clearly established that malnutrition (whether acute or chronic) is an important risk factor for malaria-related mortality [5, 10, 13, 14]. Conversely, malaria could favor the occurrence of SAM, and it has been shown that implementing malaria preventive strategies like insecticide-treated bednets improved the nutritional status of targeted populations [8].

### Relevance

Generally, studies on efficacy of antimalarial treatments follow the WHO standard protocol [15] in which children with SAM are systematically excluded. Consequently, the efficacy of antimalarial drugs in the SAM population has been poorly documented by intervention studies. Observational studies have been published regarding the efficacy of chloroquine [16], sulfadoxine-pyrimethamine (SP) [17] and cotrimoxazole [17]. Overall, efficacy of these treatments appeared to be reduced in children with SAM. Additionally, one study showed that resistance to chloroquine and SP was more frequent among malnourished children [18]. Finally, one study showed that the protective efficacy of intermittent preventive treatment with SP was impaired in malnourished children and incriminated altered pharmacokinetic (PK) profile and immune response [10].

Research on the efficacy of artemisinin-combination therapies (ACTs) in malnourished children is greatly lacking. One case of treatment failure has been reported in a child with SAM treated with artemether-lumefantrine (AL) in India, without evidence of genotype resistance [19]. The authors hypothesized that the failure could be due to altered distribution and increased clearance of the drug, though PK measurements were not performed. The first longitudinal efficacy study in malnourished children compared AL and dihydroartemisinin-piperaquine and reported good efficacy (less than 1 % failure rate) [20]. A second recently published study compared the efficacy of artesunate-amodiaquine (ASAQ) combination in DRC between SAM and non-SAM children [21], and did not show evidence for decreased efficacy in children with SAM. However, the SAM children had lower baseline parasitemia. Of interest, none of these two studies measured drug concentrations.

In the population of malnourished children, specific PK properties might alter the efficacy of antimalarials. Malnutrition is associated with increased total body water leading to increased volume of distribution of drugs [22]. Moreover, intestinal malabsorption and villous atrophy are frequent [23]. Both mechanisms contribute to lower drug concentrations. Few PK studies of antimalarials were conducted in malnourished children. They all concerned chloroquine and quinine, and indeed showed that the concentration achieved might be sub-optimal [24–27]. The PK profile of ACTs has never been assessed in children with SAM. In particular, no study has been performed regarding the PK of AL, which is currently the first-line regimen most commonly used globally including in high burden Sub Saharan countries.

Further studies evaluating the effect of malnutrition on ACTs efficacy are warranted. These studies should also assess PK of ACTs in severely malnourished children. Unless drug concentrations are measured, it is impossible to distinguish clinical treatment failure resulting from an inadequate drug exposure from failure due to drug-resistant parasites.

The MAL-NUT study aims to address the lack in knowledge regarding the efficacy and PK of AL in children with SAM. AL is becoming even more widely used as first line treatment recommended in Sahel countries, with the implementation of malaria seasonal chemoprophylaxis with SP plus Amodiaquine precluding the use of artesunate-amodiaquine and artesunate-SP in these areas.

### Hypothesis and aims

The research question is: Is the current recommended dose of AL for the treatment of uncomplicated malaria less efficacious in the children with severe acute malnutrition (SAM children) compared to the non-SAM children and is PK in cause? We hypothesize that AL efficacy might be impaired in SAM children, due to altered lumefantrine PK profile in this population.

The study aims to assess whether the current treatment dose is adequate for SAM children. It is expected that results will inform further recommendations for malaria treatment in this important target population.

The primary objective of the study is to compare the rates of adequate clinical and parasitological response (ACPR), after PCR correction, between SAM and non-SAM children, during 28 days of follow-up.

The secondary objectives are:

- To compare the day 7 lumefantrine concentration and PK profile of lumefantrine between SAM and non-SAM children (The PK of artemether will not be assessed due to feasibility concerns)
- To assess the effect of malnutrition on i) therapeutic efficacy, ii) PK profile of lumefantrine, with adjustment for other cofactors
- To compare the time to parasite clearance between SAM and non-SAM children
- To compare the rates of different types of failure (early treatment failure (ETF), late clinical failure (LCF), late parasitological failure (LPF)) between SAM and non-SAM children;
- To evaluate the relationship between treatment failure and the concentration of lumefantrine in the blood;
- To compare the rates of reinfection and recrudescence during a follow-up period of 42 days between SAM and non-SAM children; and
- To compare the nature and frequency of adverse events between SAM and non-SAM children.

As an exploratory objective, we also propose to evaluate the effect of malnutrition on the acquisition of specific antimalarial immunity. Malnutrition is known to cause relative immunosuppression, but very few studies documented its effect on specific antimalarial antibodies. We will compare the level of anti-AMA-1 antibodies at baseline between SAM and non-SAM children, after adjustment for age.

## Methods/design

### Study design and sample size

MAL-NUT is a non-randomized non-blinded comparative trial designed to compare the efficacy of AL and the lumefantrine pharmacokinetic profile in SAM versus non-SAM children. The two groups to be compared will not be randomly allocated since they include different populations of children. Similarly, blinding is not applicable.

Enrolled children will receive AL orally at the WHO-recommended weight-based dose over 3 days under directly observed treatment, and will be followed prospectively for 42 days according to the standardized WHO methods for surveillance of antimalarial drug efficacy in high transmission settings [15].

Follow-up will allow detecting early and late clinical or parasitological failure, with PCR genotyping to distinguish recrudescence from reinfection. The lumefantrine PK profile will be assessed using a population-based approach to restrict the number of samples required per child.

Sample size calculations were designed to detect a minimum crude difference in the proportion of adequate clinical and parasitological response (ACPR) between SAM and non-SAM children, with a ratio of 1/2 between the two groups (to ease the recruitment of SAM children). A total number of 540 children (180 SAM and 360 non-SAM) would allow to detect a minimum difference of 8 % in ACPR proportion (87 % in SAM versus 95 % in non-SAM children), with a statistical power of at least 80 % and a 2-sided significance level of 5 %, and 15 % expected missing endpoints. Two thirds of the children will be enrolled in Mali during the 2013 and 2014 malaria seasons, and one third will be enrolled in Niger during the 2014 malaria season.

### Settings

The MAL-NUT study will be conducted in two sites, Oulessebougou in the region of Koulikoro and Maradi in the district of Maradi, located in in the south of Mali and Niger respectively. In both countries, malaria transmission is seasonal with an annual peak of high transmission during and shortly after the rainy season, usually between July and December. AL is one of the recommended first-line malaria treatments. Since 2012, seasonal malaria chemoprevention (SMC) with SP and amodiaquine has been adopted by both countries and is being implemented in several areas (including 4 of the 14 sub districts of the Ouelessebougou district in Mali, and the Madarounfa district neighboring the Maradi district in Niger), but not in the areas were enrolments will be conducted.

Regarding malnutrition, despite progress its prevalence remains at the emergency level in Mali, and Niger remains the country the most affected worldwide [28]. In the Koulikoro region of Mali, the prevalence of global acute malnutrition (moderate or severe) in children under five was estimated to 8.6 % [6.7–9.5], and that of SAM to 1.8 % [1.0–2.2] in 2012 according to the country’s Demographic and Health Survey (DHS). In the region of Maradi, Niger, those figures were respectively 16.2 % [14.2–18.5] and 2.5 % [1.8–3.6] (Niger DHS 2012).

In Mali, the study will be conducted in the district hospital of Ouelessebougou, located in the Koulikoro region, about an hour’s drive from Bamako. This hospital hosts the Malaria Research and Training Centre (MRTC) for clinical research activities, and a nutritional rehabilitation program conducted by the Medical Alliance Against Malaria.

In Niger, children will be recruited in the primary care health center of Andoumè in Maradi city, where malnutrition activities are supported by UNICEF. In Maradi, Epicentre has a permanent research center that has been collaborating with the Ministry of Health on malnutrition and malaria research for many years including clinical trials.

### Participants

The study population will consist of children aged 6–59 months with uncomplicated *Plasmodium falciparum* malaria (confirmed by thick and thin blood films), and whose parents or guardians have given their free and informed written consent to participate in the study.

#### Eligibility criteria

A child will be eligible to be enrolled in the study if he/she meets all the inclusion criteria listed below:

- Age between 6 and 59 months
- Weight ≥ 5 kg
- Axillary temperature ≥ 37.5 °C or history of fever during the previous 24 h as reported by the parent/guardian
- *P. falciparum* monoinfection confirmed on blood film
- Parasitic density between 1,000 and 200,000 asexual forms/μL of blood.
- High probability of compliance with follow-up visits (no near-term travel plans)
- Written consent of a parent or guardian who is at least 18 years of age
- According to the group: in SAM children, weight-for-height z-score < -3 SD or MUAC <115 mm and/or bilateral edema, and in non-SAM children, weight-for-height z-score ≥ - 3 SD, and MUAC ≥ 115 mm and absence of edema.

A child will not be eligible if at least one of the exclusion criteria listed below is present:

- General danger signs or signs of severe malaria as defined by the WHO;
- Mixed or mono-infection with another *Plasmodium* species detected by microscopy;
- Severe anemia (hemoglobin <5 g / dL);
- Known underlying chronic or severe disease (e.g. HIV/AIDS, TB, cardiac, renal or hepatic disease, sickle cell);
- Presence of febrile conditions due to diseases other than malaria which could alter the outcome of the study;
- Known history of hypersensitivity or contra-indication to any of the study medications: artemether, lumefantrine (first-line medications), or artesunate, amodiaquine (rescue medications)History of a full treatment course with AL in the past 14 days
- Height-for-age < -3 Z scores (severe chronic malnutrition)
- Severe complications of malnutrition requiring hospitalization in intensive care or stabilization

### Screening and enrolment

The definition of severe acute malnutrition is a weight-for- height < -3 Z score according to the WHO standards (2006) and / or MUAC less than 115 mm and/or presence of bilateral edema [4]. Children meeting this definition will be recruited in the SAM group; non-severely malnourished children without exclusion criteria will be enrolled in the non-SAM group.

#### Screening

To recruit SAM children, children presenting at the nutritional center, meeting the following criteria and interested to participate in the study will be referred to the study staff: Aged 6 to 59 months, SAM according to WHO definition [4], fever or history of fever, no danger signs, positive malaria HRP2 rapid diagnostic test (RDT). The study staff will then explain to the parent or a guardian over 18 years about the overall study objectives, procedures and obligations attached to the study participation. If he/she agrees, a capillary blood sample will be taken from a finger prick for thick blood film microscopy and hemoglobin concentration assessments.

Regarding the enrolment of non-SAM children, malaria cases will be sought at the routine pediatric consultation. Children with fever or a history of fever and positive RDT who are interested to participate into the study will be referred to the study staff and after informed consent is obtained, a capillary blood sample will be taken from a finger prick for thick blood film and measurement of hemoglobin concentration. Enrolments will be limited to the first two eligible non-SAM children received after the enrolment of a SAM child (ideally within an interval of one week), to allow carrying out inclusions in parallel for the two study groups, with benefit in terms of representativeness of the study population throughout the malaria season.

#### Enrolment

Screening blood films will be read on-site. If the parasite density is between 1000 and 200 000 parasites per microliter, the child will be referred to the clinical investigator who will complete the eligibility screen. Eligible patients will be enrolled in the study and assigned an identification number after written informed consent has been obtained.

### Intervention

#### Antimalarial treatment under evaluation

The treatment under evaluation is artemether 20 mg - lumefantrine 120mg: Coartem® Novartis (Basel, Switzerland) will be administered six times, twice daily for 3 days (1 tablet per intake for bodyweights between 5 and 14.99 Kg; and 2 tablets for those weighing between 15 and 24.99 Kg).

The interval between the first and second administration is 8 +/-1 h, then subsequent intakes will be administered in the morning and evening, 12 h +/- 2 h apart, i.e. 24, 36, 48 and 60 h after the first dose. To improve absorption, treatment intake will be associated with fat intake. In non-SAM children, the tablets should be administered with a glass of milk, or in the middle of a breastfeeding. In SAM children, treatment should be administered with ready-to-use therapeutic food (RUTF) or therapeutic milk.

All AL doses will be directly observed by the study nurse. Patients will then remain under observation for 30 min. If vomiting occurs, a second dose should be administered. If the child vomits after the second dose, he will be removed from the study and rescue treatment will be administered.

#### Additional treatments and medications

All children will receive an insecticide-treated bed net at enrolment.

One tablet of 5 mg of folic acid will be administered to each child at Day 0. A systematic deworming by albendazole will be administered at Day 14, according to national guidelines. If the axillary temperature exceeds 38.5 °C and external cooling measures are insufficient, a dose of 15 mg/kg of paracetamol can be administered and repeated every 6 h. Iron supplementation will be administered in case of anemia for children not requiring nutritional supplements (otherwise, iron is already included in nutritional supplements).

Additionally in SAM children, feeding medications and associated therapies will be provided according to the national nutrition programs of Mali and Niger: RUTF (Plumpynut®) will be provided, corresponding to an intake of approximately 170 kcal/kg/day. The child will be closely monitored, if his nutritional status does not improve or if he encounters a medical complication, he will be transferred to the intensive care unit. A systematic treatment with oral amoxicillin will be administered for 7 days, twice a day at the dosage of 50 to 100 mg/Kg/day. A dose of vitamin A will be administered at Day 28, except for children who have already received a dose in the previous 4 months. Finally, SAM children without prior vaccination will be vaccinated against measles at Day 28.

Other drugs may be administered for concomitant diseases. However, antibiotics with antimalarial activity will be prohibited for the duration of follow-up, unless no alternative treatment is available.

#### Rescue treatment

Antimalarial rescue treatment will be administered in case of treatment failure, repeated vomiting or malaria infection with species other than *P. falciparum*.

In case of treatment failure without signs of severe malaria, children will receive Artesunate-Amodiaquine fixed combination (ASAQ Winthrop®, Geneva, Switzerland) orally, which is the second-line treatment in Mali and Niger, administered at fixed hours for 3 days (25 mg AS /67.5 mg AQ under 9 Kg, 50 mg/135 mg between 9 and 18 Kg, 100 mg/270 mg above 18 Kg).

In case of treatment failure with signs of severe malaria or oral administration impossible (repeated vomiting), artesunate will be administered IV at a dose of 2.4 mg / kg at 0, 12 and 24 h, and then every 24 h until the patient can tolerate oral treatment. An alternative is the use of artemether IM at a dose of 3.6 mg/kg on day 1 followed by 1.6 mg/kg the following days. After that, a full 3-day course of ASAQ should be administered.

After rescue treatment is administered, the weekly clinical follow-up will continue until D 42 to allow the research team to verify its efficacy, and to monitor for any adverse events.

### Data collection

#### Baseline data

At baseline, socio-demographic and anthropometric characteristics, medical history, clinical data, and biological data will be collected: in addition to the blood film and hemoglobin concentration performed at screening, capillary blood will be collected from a finger prick, and a few drops will be placed on filter paper to perform PCR genotyping (all children) and to measure Anti-AMA1 IgG (sub-study restricted to children enrolled in Mali).

#### Antimalarial efficacy

Children will be kept under observation in the hospital for the 72 first hours to ensure supervised administration of the treatment. On Day 1, Day 2, Day 3, a clinical examination will be performed, axillary temperature measured, and thick and thin blood films collected from capillary blood. In addition, the determination of parasite clearance time will involve extra blood films at h6, h12, h36.

The child will return home after the study team has noted their contact information. An insecticide-treated mosquito net will be provided and the appointment schedule for weekly follow-up visits will be clearly explained to the parent/caretaker.

On day 7, 14, 21, 28, 35 and 42, the child will undergo a physical examination with temperature measurement, and thick and thin blood films from capillary blood. Adverse events and concomitant medications will be reported. The measurement of hemoglobin concentration will also be performed on day 28.

If the child attends the study center for an unscheduled visit, a physical examination will be performed. If symptoms are suggestive of malaria, blood films will be performed.

In case of treatment failure, capillary blood will be collected on filter paper for PCR genotyping and lumefantrine concentration assay and the rescue treatment will be administered.

Children not attending their appointment will be actively traced by home visitors the next morning.

For children with SAM, each weekly visit in the study will be combined with a visit to the nutritional rehabilitation center for monitoring and treatment of malnutrition.

**Fig. 1:**
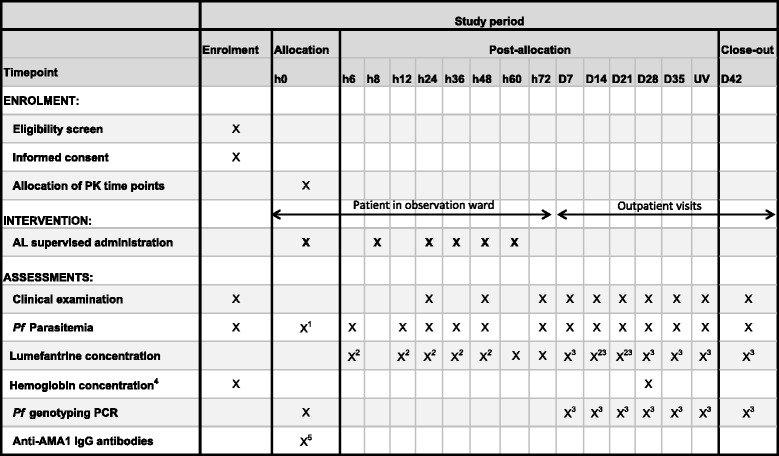
Schedule of follow-up activities. UV, unscheduled visit ; PK, pharmacokinetics ; AL, artemether-lumefantrine ; Pf, Plasmodium falciparum. ^1^ if screening parasitemia performed more than 2 h before h0. ^2^ first and last PK sampling times randomly allocated (first: h6 or h12 or h24 or h36 or h48; last: day 14 or day 21). ^3^ In case of treatment failure, additional capillary sampling for PCR genotyping and concentration of lumefantrine. ^4^ Hemoglobin concentration assessed at any point during follow-up if deemed necessary according to clinical condition. ^5^ Restricted to patients enrolled in Mali

The schedule of follow-up activities is summarized in Fig. 1.

#### Pharmacokinetics

Lumefantrine concentrations will be analysed using a population-based approach to restrict the number of samples required per child [29]. To achieve adequate precision in the estimation of PK parameters, assays will be conducted in 150 SAM and 150 non-SAM children. SAM children participating in the PK analysis will be the first consecutive patients recruited in both study sites, and non-SAM will be those recruited just after each SAM case.

For ethical and practical reasons, wherever possible, we will combine the PK samplings with other samplings performed for assessing parasitological efficacy. PK modelers have the following population sampling scheme: The first collection will be performed at h6, h12, h24, h36 or h48 (time randomly allocated), the second at h60 (the evening of day 2), the third at h72 (just before the discharge), the fourth at day 7, and the fifth at day 14 or day 21 (randomly allocated). An additional dosage will be made on the day of failure, if parasitological failure occurs.

The random allocation sequence of PK sampling times will be computer-generated by Excel before the study starts. The randomization list with be retained in each study site by the investigators, who will directly implement the allocation sequence (no blinding is required).

The PK of artemether and its active metabolite, dihydroartemisinin, will not be performed due to the heavy burden in this vulnerable population as well as logistical concerns as three venous blood samplings between h0 and h8 then plasma storage and shipment at -80 °C would be required. As an alternative, the parasite clearance will be determined in all patients, as it is considered the best indicator of impaired efficacy of the artemisinin component of an ACT [30]. A prolonged parasite clearance in children with SAM would support an impaired efficacy of artemether, one possible cause being inadequate exposure to artemisinins. On the other hand, an unaltered clearance would suggest that artemisinin exposure and efficacy is adequate. The Data Safety Monitoring Board (DSMB) will have the responsibility to monitor closely parasite clearance data, and to recommend a protocol amendment adding artemether PK assessment if deemed necessary.

### Laboratory methods

During this study, only capillary blood from finger pricks will be collected. The total amount of blood required for each patient included will approximate 7 ml for the total duration of follow-up.

For malaria screening, the SD Bioline® HRP2 RDT (Gyeonggi-do, Republic of Korea*)* will be used.

Thick and thin blood films will be read using a 100x objective. A sample will be considered negative only after 200 microscopic fields have been evaluated. The number of *P. falciparum* asexual forms on the thick film will be counted using the technique recommended by the WHO against at least 200 leukocytes [31]. Parasite density will be calculated based on a hypothetical leukocyte density of 8000 leukocytes/μL. The presence of gametocytes will also be assessed. For internal quality control during the study, a blinded second evaluation of the slides will be conducted by a second microscopist and in case of important deviation between the two readings, the procedures will be revised. An external quality control will be carried out by an external laboratory periodically during the study, for a random sample of 10 % of the positive and negative films.

Hemoglobin concentration will be determined with a HemoCue B®-Hemoglobin brand device (Ängelholm, Sweden) on day 0 and day 28.

PCR genotyping of malaria parasites will be performed to differentiate between a recrudescence and a re-infection. For this purpose, samples will be collected at enrolment and at treatment failure (if any) on filter paper and stored individually in re-sealable, zippered pouches containing a desiccant and sheltered from moisture, excessive heat and light. PCR genotyping will be carried out at the MRTC laboratory in Bamako. PCR adjustment consists of comparing primary infection parasites with those responsible for secondary infections. Identical genotypes at the *glurp*, *msp-2* and *msp-1* loci are a sign of treatment failure, while distinct genetic profiles indicate reinfection [32]. The paired pre- (day 0) and post-treatment (day X) samples with similar genotypes will be classified as a recrudescence (true failures), while pairs with different genotypes will be classified as re-infections. This analysis will also allow the complexity of infection (single or multiple) to be determined.

For lumefantrine concentration assessment, capillary blood will be collected in a microcapillary tube coated with lithium heparinate, then immediately transferred to a filter paper previously treated by the laboratory of Cape Town (50 microliter of whole blood per spot). Once dry, the filter papers will be stored away from heat and excessive light and regularly sent to the Division of Clinical Pharmacology, University of Cape Town for analysis. Quality controls spiked by the analytical laboratory, specifically designed to control for the environmental conditions at the site and during storage, will be stored and sent under the same conditions. PK assays will be performed using liquid chromatography and tandem mass spectrometry (LC MS/MS).

At h0, for children enrolled in Mali, 3 drops of capillary blood will be placed on filter paper for the dosage of anti-AMA1 specific antimalarial antibodies by Elisa method. Analysis will be performed by MRTC, Bamako.

### Outcome measures

The primary outcome of this study is the proportion of patients having an adequate clinical and parasitological response (ACPR) as defined by the WHO [14] on day 28, after PCR correction. This proportion will be compared between SAM and non-SAM children. Kaplan-Meier analysis will be used to account for censored data.

Secondary outcomes are:

- Regarding treatment efficacy
  - ○ Corrected ACPR proportion on day 42
  - ○ Failure proportion by type (ETF, LCF, LPF) by day 28 and 42, as defined by WHO [14]
  - ○ Proportion of reinfection and recrudescence
  - ○ Time to parasite clearance and parasite clearance half-life
- Regarding PK of lumefantrine
  - ○ Area under curve (AUC) and other PK parameters (Cmax, Tmax, Clearance, Volume of Distribution)
  - ○ Concentration on day 7
  - ○ Concentration on day of failure (in case of failure)
- Regarding safety
  - ○ Type and frequency of adverse events

### Protocol deviations and withdrawals

Children will be followed until day 42 whenever possible. However, some reasons may lead to the interruption of monitoring (such as protocol deviation, study withdrawal, detection of a malaria infection with species other than *P. falciparum*, death, severe adverse event, loss to-follow-up…). In all cases, the date and reasons for the interruption will be collected in the report form.

### Adverse events

Safety and tolerability of AL have been previously assessed and the treatment has been recommended for routine clinical use. The summary of product characteristics of study drugs will be available to clinicians to help the assessment of all adverse events and their relation with study drugs.

Any clinical or laboratory adverse event occurring during the 42-day follow-up after dosing will be described by the investigator in a specific section of the case report form. The following information will be collected: date of onset, nature, severity graded on a scale from 1 to 4 (minor, moderate, severe, and life-threatening), seriousness, relationship with AL, action(s) taken, date of resolution, outcome.

A physician investigator will be available on site 24h per day for the management of adverse events. In case of a SAE, as defined by ICH (ICH, CIOMS, European directive 2001/CE/21), a specific SAE declaration form will be filled in by the site principal investigator within 48 h and sent to the investigator coordinator in Epicentre, the Ethics Committees and the DSMB. They will recommend any action to be taken (including early termination of the study). In addition, ethics committees who reviewed and approved the protocol will also receive periodical reports of aggregated data on AEs, at regular intervals throughout the study.

### Statistical considerations

Study data will be double entered using REDCap electronic data capture tools hosted at Epicentre [33] then data management and analysis will be performed with STATA 12, StataCorp®, College Station, TX, according to written data entry procedures and data management plan.

A flowchart will present the following information: number of patients screened, number of patients infected with *P. falciparum* malaria, number of patients infected by other *Plasmodium* species, number of patients excluded (and reasons why they were not eligible), number of patients enrolled and analyzable in each group of SAM and non-SAM children.

For all enrolled children, baseline characteristics will be presented for each group (SAM versus non-SAM).

#### Antimalarial efficacy

Clinical and parasitological efficacy outcomes will be presented for SAM and non-SAM children: number of ETF, LCF, LPF, ACPR, re-infection, recrudescence, mixed infection, by day 28 and day 42.

Two methods will be used to estimate the therapeutic efficacy in each group:

- The Kaplan-Meier survival analysis will be used to calculate estimates of survival without subsequent infection in both intent-to-treat and per-protocol populations. This analysis will allow accounting for censored data [15, 34].
- Logistic regression analysis will be used to compare at day 28 (and then day 42) the proportion of ACPR in patients for which the endpoint has been determined, excluding those lost to follow-up or with missing endpoint.

After univariate analyses, multivariate analyses using survival models (time to recurrence) and logistic regression (proportion of ACPR) will be performed where the therapeutic success will be explained by the degree of malnutrition and other cofactors, including lumefantrine PK profile.

#### Estimation of the PK profile of lumefantrine

PK modeling will estimate the lumefantrine area under the curve, Cmax, Tmax, Volume of Distribution and Clearance in SAM and non-SAM children using nonlinear mixed effect models (NONNEM).

The values obtained will be compared between groups, and related to parasitological efficacy.

#### Estimation of parasite clearance

Regression models of the log-transformed parasite counts will be fitted in order to estimate parasite clearance using the Parasite Clearance Estimator Tool (PCE) developed by the WorldWide Antimalarial Resistance Network (WWARN) [30]. Parasite clearance will be compared between groups of SAM and non-SAM children on a regular basis during the study.

### Ethical considerations

A French version of the protocol and informed consent form has been approved by the Ethics Committee of the Faculty of Medicine and Odonto-Stomatologie and the Faculty of Pharmacy in Bamako, Mali and Niger National Ethics Committee of the Ministry of Health. An English version has been approved by MSF Ethical Review Board.

Any amendment to the protocol that may affect the rights, safety and / or welfare of the participants or the conduct of research will be submitted again to Ethics Committees before being implemented.

Individual data will not be disclosed to anyone outside the research team, unless it is necessary for the proper medical management of children (useful data can then be shared with the physician, in respect of medical confidentiality). The database will not contain any personally identifiable information; patients are only identified by their inclusion number.

### Monitoring and good clinical practice

The study will comply with the recommendations of Good Clinical Practice and with standards of care established by the national health programs and the Declaration of Helsinki. An external Good Clinical Practice monitor will conduct regular visits to the sites, as detailed in a dedicated monitoring plan. Data report forms will be checked on site in relation to source documents to ensure they are complete and clear. The monitor will also review compliance with written standardized operating procedures.

For this study, a DSMB will be instituted including members with the following expertise: pharmacometrician, epidemiologist, malaria specialist, and pediatrician. As described in a dedicated DSMB charter, it will be responsible for regularly review the results regarding the enrolment of patients, and the efficacy and safety of AL. The DSMB will advise the Scientific Committee on the continuation or, if needed the amendment or discontinuation of the trial.

### Risks and benefits for participants

Inclusion in the study is voluntary and will only be possible if the adult parent or guardian gives written informed consent.

Participants will receive a free malaria treatment and will benefit from active monitoring in the health facility for 42 days. In case of treatment failure, they will receive a rescue treatment. Concomitant diseases will also be actively sought and promptly managed. Children with SAM will benefit appropriate nutritional care. All the care provided meets the highest quality standards, and is in accordance with national guidelines.

Participants will be informed of the possible occurrence of side effects related to the study treatments, and these will be systematically recorded and managed.

The constraints for the child (and caregiver) will be to stay in the health center for the 3 days of treatment, and then return to the study site every week for 6 weeks. Reimbursements will be given in compensation for the time spent for visits and travel costs, according to the recommendations of the community leaders. During follow-up, the child will have several capillary blood collections that are not performed routinely, although the total blood volume sampled will be limited to under 10 mL so is not associated with a particular risk. The risks are fear and pain at the puncture site.

## Discussion

This study will be conducted during 2013 and 2014 malaria seasons in Mali, and 2014 malaria season in Niger. Enrolments are expected to end in early 2015 and results should be available in second semester 2015.

The study report will be discussed and circulated to the stakeholders, including the local officials. A poster summarizing the main results will be posted at the two study hospitals to inform the participants. Several oral feedback sessions will also be held on the study sites and with scientific partners and policy makers. The results will be published in scientific journals and reported at international conferences.

In the study areas, a better understanding of the efficacy of antimalarial drugs (in SAM and non-SAM children) will contribute to updating the protocols for case management of malaria, and thus contribute to improving the health of the population.

More generally, this study will provide important, lacking information on the efficacy of AL in SAM children and could inform further recommendations for the management of malaria in this specific population. It will therefore benefit all malnourished children living in malaria-endemic areas (the entire Sahel Africa is concerned). If our results indicate a lack of efficacy of AL at usual doses, we will recommend evaluating the use of increased doses through dose optimization studies.

### Declarations

**Trial status:** active recruitment
